# Serum levels of AFP and CA19-9 after intraoperative radiotherapy combined with drug therapy on liver and pancreatic tumors

**DOI:** 10.5937/jomb0-56627

**Published:** 2025-08-21

**Authors:** Xin Jia, Zongliang Jiang

**Affiliations:** 1 Zhengzhou Health Vocational College, School of Nursing, Zhengzhou, China

**Keywords:** AFP, CA19-9, intraoperative radiotherapy, medication, tumour markers, hepatopancreatic tumours, recurrence rate, AFP, CA19-9, intraoperativna radioterapija, medikamentozna terapija, tumorski markeri, hepatopankreasni tumori, stopa recidiva

## Abstract

**Background:**

To explore the efficacy of intraoperative radiotherapy combined with drug therapy on serum levels of AFP and CA19-9 for liver and pancreatic tumours to provide more effective treatment strategies for clinical practice.

**Methods:**

A retrospective analysis was conducted on 190 patients with liver and pancreatic tumours who underwent surgical resection combined with intraoperative radiotherapy in the hospital from March 2023 to September 2024. The patients were segmented into an experimental group (intraoperative radiotherapy combined with drug therapy, n = 95) and a control group (traditional treatment, n = 95) at random. After surgical resection, the experimental group accepted IORT targeted drugs, and immunomodulators. The control group received surgical resection and chemotherapy or external radiation therapy. The leading observation indicators include tumour marker levels, biochemical indicators, recurrence rate, survival rate, quality of life, postoperative complications, pain score, and psychological status.

**Results:**

The levels of AFP and CA19-9 in the experimental group decreased by 16.2 ng/mL and 74.7 U/mL, which surpassed those in the control group (P< 0.05). After treatment, the liver function indicators of the experimental group significantly improved (ALT decreased from 32 .1± 12.5 U/L to 22.4± 10.1 U/L, P = 0.00), and renal function also improved. The recurrence and metastasis rates in the experimental group were lower (P< 0.05). Although there was no discrepancy in common adverse reactions, the experimental group had a lower incidence of adverse reactions in radiation dermatitis and infection (P< 0.05). The survival curve demonstrated that the survival rate of the experimental group was higher (P< 0.05).

**Conclusions:**

Intraoperative radiotherapy combined with drug therapy has shown outstanding efficacy in treating liver and pancreatic tumours, effectively reducing tumour marker levels, improving liver and kidney function, reducing recurrence and metastasis rates, and improving survival rates and life quality while reducing postoperative complications and adverse reactions. This comprehensive treatment plan provides a new, effective strategy for treating liver and pancreatic tumours.

## Abstract (sr)

**Uvod:**

Cilj je bio da se ispita efikasnost intraoperativne radioterapije u kombinaciji sa terapijom lekovima na serumske nivoe AFP i CA19-9 kod tumora jetre i pankreasa, kako bi se obezbedile efikasnije strategije lečenja u kliničkoj praksi.

**Metode:**

Sprovedena je retrospektivna analiza na 190 pacijenata sa tumorima jetre i pankreasa koji su u bolnici, u periodu od marta 2023. do septembra 2024. godine, podvrgnuti hirurškoj resekciji u kombinaciji sa intraoperativnom radioterapijom. Pacijenti su nasumično podeljeni u eksperimentalnu grupu (intraoperativna radioterapija u kombinaciji sa terapijom lekovima, n = 95) i kontrolnu grupu (tradicionalno lečenje, n = 95). Eksperimentalna grupa je nakon hirurške resekcije primala IORT, ciljane lekove i imunomodulatore, dok je kontrolna grupa bila podvrgnuta hirurškoj resekciji i hemioterapiji ili spoljašnjoj radioterapiji. Glavni posmatrani parametri uključuju nivoe tumorskih markera, biohemijske pokazatelje, stopu recidiva, stopu preživljavanja, kvalitet života, postoperativne komplikacije, intenzitet bola i psihološko stanje.

**Rezultati:**

Nivoi AFP i CA19-9 u eksperimentalnoj grupi smanjili su se za 16,2 ng/mL i 74,7 U/mL, što je značajno više u poređenju sa kontrolnom grupom (P< 0,05). Nakon tretmana, pokazatelji funkcije jetre u eksperimentalnoj grupi značajno su se poboljšali (ALT se smanjio sa 32 ,1± 12,5 U/L na 22,4± 10,1 U/L, P= 0,00), a poboljšana je i funkcija bubrega. Stope recidiva i metastaza bile su niže u eksperimentalnoj grupi (P< 0,05). Iako nije bilo značajnih razlika u učestalosti uobičajenih neželjenih reakcija, eksperimentalna grupa je imala nižu stopu radijacionog dermatitisa i infekcija (P< 0,05).

**Zaključak:**

Intraoperativna radioterapija u kombinaciji sa terapijom lekovima pokazala je izuzetnu efikasnost u lečenju tumora jetre i pankreasa, značajno smanjujući nivoe tumorskih markera, poboljšavajući funkciju jetre i bubrega, smanjujući stope recidiva i metastaza, povećavajući stope preživljavanja i poboljšavajući kvalitet života, uz smanjenje postoperativnih komplikacija i neželjenih reakcija.

## Introduction

Hepatopancreatic tumours, including hepatocellular carcinoma and pancreatic cancer, are among the most aggressive malignancies with exceptionally high mortality rates worldwide [1]. Due to their asymptomatic progression in the early stages, many patients are diagnosed at an advanced stage, often missing the optimal window for curative treatment. Conventional therapeutic approaches, such as surgical resection, chemotherapy, and external beam radiotherapy, have shown limited efficacy, especially in reducing recurrence and improving long-term survival [2]. Therefore, developing more effective treatment strategies remains a significant challenge in clinical oncology.

Intraoperative Radiotherapy (IORT) has emerged as a promising technique, offering localised, high-dose radiation directly to tumour sites while minimising damage to surrounding healthy tissues. Recent technological advancements have further refined lORT's accuracy. For instance, Moo et al. introduced a real-time deep-learning reconstruction technique for intraoperative probes, significantly enhancing IORT precision in tumour localisation and dose distribution [3]. Similarly, Yan et al. developed a label-free tumour localisation method using stereoscopic colour fluorescence imaging, which, although originally designed for lung cancer, has potential applications in hepatopancreatic tumour treatment [4]. Moreover, the high-resolution portable gamma camera developed by Bossis et al. has provided a novel approach for evaluating absorbed radiation doses in molecular radiotherapy, improving treatment efficacy and safety [5]. These innovations highlight the growing role of IORT in oncological treatment.

While IORT offers several advantages in tumour control, its combination with drug therapy may enhance treatment outcomes further. Pharmacological agents such as targeted therapies and immunomodulators have demonstrated the potential to inhibit tumour progression and metastasis in various cancers [6]. However, most studies have focused on singlemodality treatments, and the potential synergistic effects of IORT combined with drug therapy in hepatopancreatic tumours remain underexplored [7].

This study aims to evaluate the efficacy of intraoperative radiotherapy combined with drug therapy (IORT-DT) in treating hepatopancreatic tumours, focusing on serum tumour markers such as AFP and CA19-9. By analysing tumour marker levels, biochemical indices, recurrence rates, survival outcomes, and quality of life, this research seeks to provide a more effective and comprehensive therapeutic strategy for clinical application. The novelty of this study lies in systematically investigating the combined effects of IORT and pharmacological agents as an integrated treatment approach for hepatopancreatic tumours for the first time. Through an in-depth exploration of their synergistic mechanisms, this study aspires to pave the way for more precise and personalised treatment strategies for these malignancies.

## Materials and methods

### Research objects

This retrospective study analysed 190 patients with liver and pancreatic tumours (LPT) treated between March 2023 and September 2024. Patients were randomly assigned to either the experimental group (IORT-DT, n = 95) or the control group (traditional treatment, n = 95). The study evaluated the effects of intraoperative radiotherapy combined with drug therapy (IORT-DT) compared to surgical resection with chemotherapy/external radiation therapy, focusing on tumour markers, biochemical indices, recurrence rates, survival rates, quality of life, complications, pain, and psychological status. The hospital ethics committee approved the study, and all participants provided informed consent after fully disclosing the study's purpose, process, risks, and benefits. Ethical guidelines were strictly followed to protect patient rights and data confidentiality.

### Inclusion criteria:

Patients were eligible if they met the following criteria:

1. Confirmed diagnosis of LPT, with imaging and tumour marker evaluation indicating that the tumour was resectable with the potential for complete removal.
2. No prior chemotherapy, external radiation therapy, radiofrequency ablation, or interventional therapy before surgery.
3. No history of abdominal radiation therapy and no chemotherapy within the past three months.
4. No evidence of distant metastasis based on imaging.
5. Adequate organ function, including normal cardiopulmonary, liver, kidney, and coagulation function.
6. No history of severe allergies, with an ECOG performance status suitable for surgery and follow-up compliance.

### Exclusion criteria:

Patients were excluded if they had:

1. Other malignant tumours or serious comorbidities affecting treatment outcomes.
2. Imaging or tumour marker evaluation indicating distant metastasis or the inability to achieve complete resection.
3. Prior abdominal radiation therapy or chemotherapy within the last three months.
4. Severe heart, lung, liver, kidney, or coagulation dysfunction that contraindicated surgery or IORT.
5. Severe allergies or high-risk reactions to drugs/materials used in IORT.
6. Inability to complete follow-up or provide complete treatment and evaluation data.

This study was approved by the hospital ethics committee, ensuring strict adherence to ethical guidelines. All participants provided written informed consent after being fully informed of the study's purpose, procedures, potential risks, and benefits.

### Research methods

**Figure 1:**
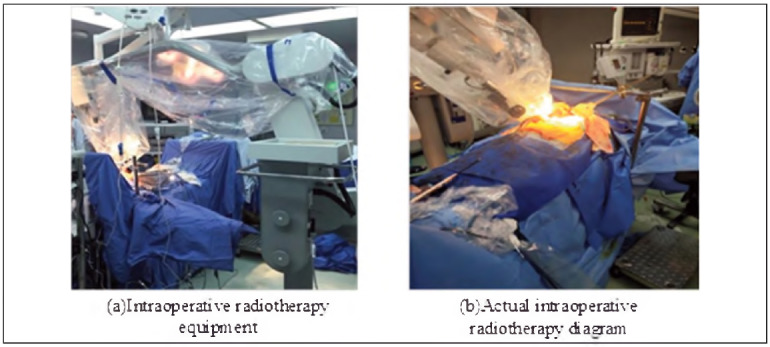
EIORT equipment and actual IORT.

To ensure the scientificity and reliability of the study, a random grouping method was utilised to divide the research subjects into the Experimental Group (IORT-DT group, EG) and the Control Group (traditional treatment group, CG). The grouping process strictly followed the randomisation principle to ensure comparability of baseline characteristics such as age, gender, tumour stage, and pathological type between the two groups. In the EG, patients will receive an IORT-DT regimen. Advanced IORT equipment was used In this treatment plan. The device Is equipped with a high-precision dose calculation system and image guidance technology, which can ensure the precise delivery of radiation and minimise damage to surrounding normal tissues. During the surgical process, after the tumour is removed or mostly removed, high-dose radiation is immediately applied to the tumour bed or residual tumour using IORT equipment [8]. Real-time deep learning reconstruction technology and label-free tumour localisation technology are used during the irradiation process to guarantee that the radiation can be delivered appropriately to the target area, further improving the accuracy and safety of treatment. Figure 1 shows the IORT device and the actual IORT diagram.

In addition to IORT, EG patients will also receive specific drug treatment regimens. Drug selection is based on the tumour's biological characteristics, the drug's anti-tumour mechanism, and the patient's physical condition [9]. The drugs mainly consist of drug combinations with synergistic effects, including targeted drugs and immunomodulators [10]. Targeted drugs can act on specific molecular targets of tumour cells, inhibiting their growth and spread. Immune modulators can enhance patients' immune function and improve the body's resistance to tumours [11]. The administration route, dosage, and duration of drug therapy are determined by considering factors such as the patient's weight, LKF, and drug metabolism to ensure the effectiveness and safety of the drug [12]. Patients with CG will receive traditional treatment methods commonly used in clinical practice. The treatment for CG patients mainly includes surgical resection, chemotherapy, or External Radiation Therapy (ERT). Surgical resection is the basis for treating the LPT. Doctors develop personalised surgical plans built on the patient's specific condition to ensure the thoroughness and safety of the surgery [13]. Chemotherapy and ERT are used as adjuvant treatments, selected and applied according to the patient's tumour stage and physical condition. The chemotherapy regimen includes chemotherapy drugs such as gemcitabine and fluorouracil, which prolong the patient's survival by inhibiting the growth and spread of tumour cells. ERT uses radiation to irradiate tumours to kill or inhibit the growth of tumour cells [14]. The treatment plan and duration for CG patients follow clinical guidelines and best practices to ensure the effectiveness and safety of the treatment.

### Observation indicators

To comprehensively evaluate the efficacy of IORT-DT in the LPT treatment, this study selected the following ten leading observation indicators: (1) Tumour Marker Level (TML): monitoring the concentration changes of Tumour Markers (TMs) such as alpha-fetoprotein (AFP) and carbohydrate antigen 19-9 (CA19-9) in the blood of patients before and after treatment [15]. (2) Biochemical indicators: Evaluate changes in biochemical indicators such as liver function (ALT, AST, ALP, total bilirubin), kidney function (blood creatinine, urea nitrogen), and electrolyte balance [16]. (3) Recurrence rate: Record the frequency and time point of tumour recurrence within a period after treatment. (4) Survival curve: Draw Kaplan-Meier survival curves to analyse the difference in survival rates between EG and CG. (5) Quality of Life (QoL): Use questionnaires such as EORTC QLQ-C30 to assess patients' QoL, including physical, role, emotional, cognitive, and social functioning [17]. (6) Postoperative Complications (POC): Record and classify POC, such as infection, bleeding, intestinal obstruction, etc. (7) Patient Pain Score (PPS): The Number Rating Scale (NRS) is used to record the degree of pain in patients [18]. (8) Psychological state: Evaluate the patient's mental health status through the Hospital Anxiety and Depression Scale (HADS) [19]. (9) Correlation analysis: Analyse the correlation between TML, biochemical indicators, QoL, and postoperative complications.

### Statistical analysis

This study uses SPSS software for data analysis to ensure the accuracy and reliability of the results. Measurement data, such as TML and biochemical indicators, are described using mean±standard deviation, and a t-test is conducted to compare the discrepancies between EG and CG. Count data, such as recurrence rate, POC incidence rate, etc., are described using frequency and percentage, and a χ^2^-test is performed. The survival curve is plotted using the Kaplan-Meier method, and the difference in survival rates between EG and CG is compared using the Log-rank test. In addition, the Spearman correlation analysis method is utilised to explore the correlation between TML, biochemical indicators, QoL, and POC. P<0.05 indicates statistical significance.

## Results

### Comparison of basic information of research subjects

To ensure comparability of baseline characteristics between EG and CG patients, this study conducted statistical analysis and comparison of age, gender, tumour stage, pathological type, and other relevant baseline data between the two groups. Table 1 shows the specific results. There was *P*>0.05 in baseline information like age, gender, weight, BMI, tumour stage, pathological type, preoperative AFP level, preoperative CA19-9 level, liver function, and kidney function between EG and CG patients (*P*>0.05). This indicates that these baseline characteristics are comparable, providing a reliable basis for subsequent efficacy evaluation.

**Table 1:** Comparison of baseline characteristics between EG and CG.

Baseline feature	EG (n=95)	CG (n = 95)	χ^2^/t	*P*
Age (years)	58.3±10.5	57.9±11.2	0.30	0.76
Gender (Male/female)	56/39	58/37	0.11	0.72
Weight (kg)	65.8±12.3	66.2 ± 11.9	-0.25	0.80
BMI (kg/m^2^)	24.1 ±3.5	24.3±3.7	-0.41	0.69
Tumour stage (l/ll/lll/IV)	12/35/38/10	10/37/39/9	1.54	0.85
Pathological type (hepatocellular<br>carcinoma/pancreatic cancer)	60/35	58/37	0.09	0.78
AFP level (ng/mL)	28.5±6.1	29.1 ±6.4	-0.19	0.87
CA19-9 level (U/mL)	120.5±18.7	118.9±21.3	0.12	0.91
ALT	32.1 ± 12.5	31.3 ±11.8	1.25	0.75
AST	35.5±13.2	34.2±12.7	1.56	0.78
ALP	98.2±25.6	97.7±24.9	1.00	0.81
Total bilirubin	0.9±0.3	0.8±0.2	1.96	0.67
Serum creatinine	0.9±0.2	0.8±0.2	2.00	0.65
Urea nitrogen	6.8±1.9	6.9±1.8	-0.33	0.79

### Comparison of TML changes

**Figure 2:**
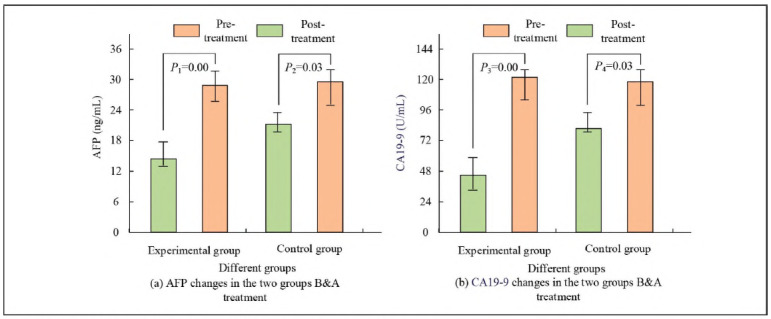
Changes of TML in EG and CG before and after treatment.

To evaluate the therapeutic effect of IORT-DT on LPT, the changes in TML before and after treatment were compared between EG and CG patients, as shown in Figure 2. The AFP level of EG decreased by 16.2 ng/mL, while CG only reduced by 8.6 ng/mL. The CA19-9 level of EG decreased by 74.7 U/mL, and CG decreased by 39.9 U/mL. The decrease in EG's TML was more significant than in CG (*P*<0.05).

### Comparison of changes in LKF indicators

To comprehensively assess the effects of IORT-DT on liver function, kidney function, and electrolyte balance in patients with hepatopancreatic tumours, the changes in serum indicators of B&A treatment were compared between EG and CG patients, as shown in Table 2. After treatment, significant statistical differences (*P*<0.05) were observed in the ALT, AST, ALP, total bilirubin, serum creatinine, and urea nitrogen levels of EG compared to CG. EG patients' liver function indicators (ALT, AST, ALP, total bilirubin) decreased after treatment, while renal function indicators (serum creatinine, urea nitrogen) also improved.

**Table 2:** Changes of serological indexes in EG and CG B&A treatment.

Indicator	Time point	EG (n=95)	CG (n=95)	t	*P*
ALT (U/L)	Pre-treatment	32.1 ±12.5	31.3 ± 11.8	1.25	0.75
	Post-treatment	22.4±10.1	28.6±11.5	4.37	0.00
AST (U/L)	Pre-treatment	35.5±13.2	34.2±12.7	1.56	0.78
	Post-treatment	2 3.7 ± 11.0	30.1 ±12.4	4.02	0.00
ALP (U/L)	Pre-treatment	98.2±25.6	97.7 ±24.9	1.00	0.81
	Post-treatment	76.4±23.1	89.3±24.3	4.15	0.00
Total bilirubin<br>(μmol/L)	Pre-treatment	0.9±0.3	0.8±0.2	1.96	0.67
	Post-treatment	0.6±0.2	0.7±0.3	2.58	0.00
Serum Creatinine<br>(μmol/L)	Pre-treatment	0.9±0.2	0.8±0.2	2.00	0.65
	Post-treatment	0.8±0.2	0.9±0.2	3.54	0.00
Urea nitrogen<br>(mmol/L)	Pre-treatment	6.8±1.9	6.9±1.8	-0.33	0.79
	Post-treatment	5.2±1.7	6.5±1.9	5.12	0.00

### Analysis of recurrence rate

**Figure 3:**
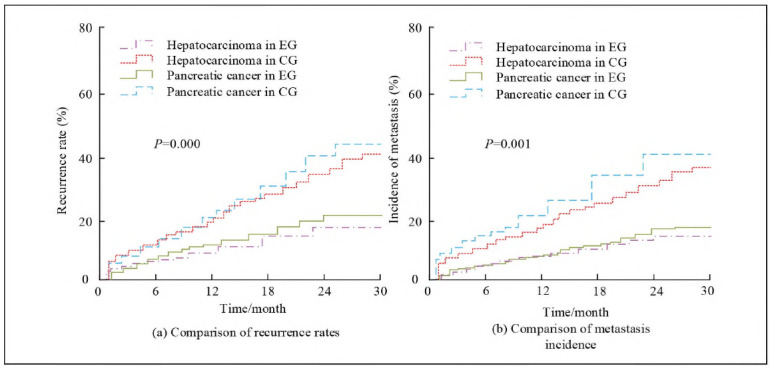
Comparison of recurrence rate and incidence of metastasis between the EG and the CG at different time points after treatment.

To evaluate the effect of IORT-DT on the recurrence of LPT, EG and CG patients' recurrence and metastasis rates at different time points after treatment were compared, as shown in Figure 3. In Figure 3 (a), during the follow-up period, the recurrence rate of EG remained stable and relatively low over time, while the recurrence rate of CG showed a more significant upward trend. The difference in recurrence rate between the two groups is noticeable (*P*<0.05). In addition, it could be observed from Figure 3 (b) that the metastasis incidence in EG was lower than CG (*P*<0.05).

### Comparison of adverse reactions

To comprehensively evaluate the safety of IORT-DT, the Adverse Reactions (ARs) that occurred during and after treatment in EG and CG patients were compared. AR classification was based on the Common Terminology Criteria for Adverse Events (CTCAE) standard, and the specific results are listed in Table 3. Table 3 listed the common types of ARs and their incidence rates in each group, including the severity grading of ARs (grades 1 to 3, with grade 1 being the lightest and grade 3 being the heaviest). Although there was *P*<0.05 in the incidence of common ARs like nausea, vomiting, diarrhoea, fatigue, neutropenia, and anaemia among patients (*P*>0.05), the AR incidence in radiation dermatitis and infection in EG was lower. Meanwhile, EG also showed a lower trend in the Wound Healing Complications (WHC) incidence, although this difference did not reach a statistically significant level (*P*=0.08).

**Table 3:** Comparison of ARs between EG and CG.

AR	Severity Grade	EG (n = 95)	CG (n = 95)	χ^2^	P
Nausea and<br>Vomiting	1	28 (29.5%)	32 (33.7%)	2.31	0.68
	2	12 (12.6%)	16 (16.8%)		
	3	3 (3.2%)	5 (5.3%)		
Diarrhea	1	19 (20.0%)	24 (25.3%)	3.02	0.55
	2	8 (8.4%)	12 (12.6%)		
	3	2 (2.1%)	3 (3.2%)		
Fatigue	1	35 (36.8%)	40 (42.1%)	2.04	0.84
	2	15 (15.8%)	18 (18.9%)		
	3	6 (6.3%)	7 (7.4%)		
Neutropenia	1	11 (11.6%)	15 (15.8%)	4.52	0.34
	2	7 (7.4%)	10 (10.5%)		
	3	4 (4.2%)	6 (6.3%)		
Anemia	1	18 (18.9%)	22 (23.2%)	3.78	0.44
	2	10 (10.5%)	14 (14.7%)		
	3	4 (4.2%)	5 (5.3%)		
Radiodermatitis	1	3 (3.2%)	0 (0.0%)	10.45	0.01
	2	5 (5.3%)	0 (0.0%)		
	3	2 (2.1%)	0 (0.0%)		
Infection	Any grade	7 (7.4%)	14 (14.7%)	3.94	0.04
WHC	Any grade	4 (4.2%)	9 (9.5%)	3.06	0.08

To assess the IORT-DT effect on POC in LPT patients, comprehensive records and statistics were conducted on complications that occurred in EG and CG patients after surgery. The classification of POC was based on the internationally recognised surgical complication classification standards, taking into account the specificity of liver and pancreatic tumour surgery, as shown in Table 4. Table 4 presented the common POC types and incidence rates in each group, including the severity grading of complications (mild, moderate, severe). EG had a significantly lower overall incidence of complications than CG (*P*=0.01), meaning that the IORT-DT may help reduce POC in patients with LPT. Although the discrepancy between the two groups did not reach statistical meaning in certain specific types of complications (*P*>0.05), EG showed a lower trend in the occurrence of common complications like infection, bleeding, and pancreatic fistula.

**Table 4:** Comparison of POC between EG and CG.

Complication Type	Severity Grade	EG (n = 95)	CG (n = 95)	χ^2^	*P*
Infection	Mild	12 (12.6%)	18 (18.9%)	4.27	0.23
	Moderate	4 (4.2%)	8 (8.4%)		
	Severe	1 (1.1%)	3 (3.2%)		
Bleeding	Mild	7 (7.4%)	10 (10.5%)	4.56	0.10
	Moderate	2 (2.1%)	5 (5.3%)		
	Severe	0 (0.0%)	1 (1.1%)		
Anastomotic Leak	Mild	3 (3.2%)	5 (5.3%)	0.67	0.88
	Moderate	2 (2.1%)	3 (3.2%)		
	Severe	0 (0.0%)	1 (1.1%)		
Pancreatic Fistula	Mild	5 (5.3%)	9 (9.5%)	1.89	0.39
	Moderate	3 (3.2%)	5 (5.3%)		
	Severe	1 (1.1%)	2 (2.1%)		
Bile Leak	Mild	4 (4.2%)	6 (6.3%)	0.44	0.80
	Moderate	1 (1.1%)	2 (2.1%)		
	Severe	0 (0.0%)	0 (0.0%)		
Delayed Gastric Emptying	Mild	6 (6.3%)	10 (10.5%)	1.07	0.78
	Moderate	3 (3.2%)	4 (4.2%)		
	Severe	0 (0.0%)	1 (1.1%)		
Chyle Leak	Any grade	1 (1.1%)	3 (3.2%)	1.03	0.31
Overall Complication Rate	Any grade	43 (45.3%)	58 (61.1%)	6.54	0.01

**Figure 4:**
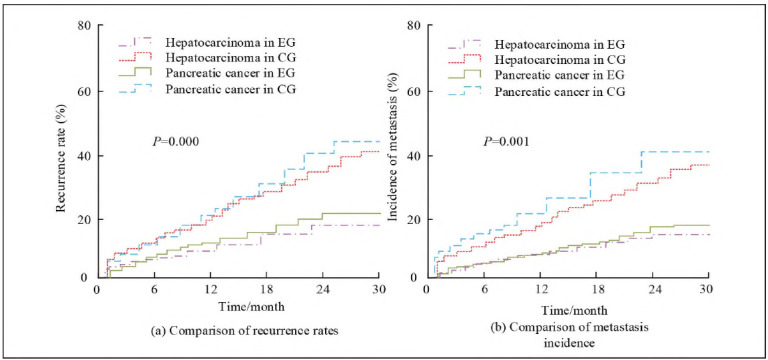
Comparison of mortality and survival rates of patients with hepatopancreatic tumours between the two groups.

To evaluate the effect of IORT-DT on the survival time of LPT patients, survival curves were plotted for two groups of patients, as shown in Figure 4. The survival curves of patients with LPT in EG were all above CG, and the death curves were all below CG, indicating that the survival rate of EG was higher. The Logrank test further confirmed the *P*<0.05 in survival rate between the two groups, supporting the effectiveness of this comprehensive treatment plan.

### QoL assessment

To comprehensively evaluate the impact of IORT-DT on the QoL of patients with LPT, EORTC QLQ-C30 and other questionnaires were used to assess B&A treatment. The evaluation covered multiple dimensions: physical function, role, emotion, cognition, and social function (Table 5). In pre-treatment, there was *P*>0.05 in QoL scores between patients with EG and CG in various dimensions. However, in post-treatment, the scores of various functional dimensions of EG surpassed the CG (*P*<0.05), indicating that combining IORT and drug therapy can help improve the QoL of patients with LPT

**Table 5:** Comparison of QoL.

QoL Dimension	Time Point	EG (n=95)	CG (n=95)	t	*P*
Physical Functioning	Pre-treatment	75.2±15.8	74.9±16.3	0.12	0.90
	Post-treatment	82.4±12.6	76.8±14.1	3.14	0.00
Role Functioning	Pre-treatment	68.3±20.5	67.9±21.2	0.14	0.89
	Post-treatment	78.1 ±17.3	70.5±19.6	2.89	0.00
Emotional Functioning	Pre-treatment	65.4±18.7	64.8±19.1	0.21	0.83
	Post-treatment	72.6±16.2	66.9±17.8	2.36	0.02
Cognitive Functioning	Pre-treatment	80.1 ±14.2	79.6±14.8	0.25	0.80
	Post-treatment	84.5±11.9	80.2±13.5	2.17	0.03
Social Functioning	Pre-treatment	72.5±17.6	71.8±18.1	0.27	0.79
	Post-treatment	80.9±15.3	74.6±16.9	2.71	0.01
Fatigue	Pre-treatment	34.5±21.3	35.1 ±20.8	0.18	0.86
	Post-treatment	28.7±18.6	32.9±19.5	1.46	0.15
Pain	Pre-treatment	32.6±24.1	33.2±23.8	0.15	0.88
	Post-treatment	23.1 ±19.5	28.4±21.3	1.79	0.07
Dyspnea	Pre-treatment	18.9±22.5	19.5±21.9	0.17	0.86
	Post-treatment	16.7±20.1	18.8±21.2	0.65	0.52
Insomnia	Pre-treatment	29.8±26.3	30.4±25.8	0.14	0.89
	Post-treatment	25.1 ±23.7	28.9±24.6	1.07	0.29
Constipation	Pre-treatment	15.9 ± 21.1	16.4±20.7	0.14	0.89
	Post-treatment	14.3±19.8	17.2±20.3	0.92	0.36

### Correlation analysis

**Figure 5:**
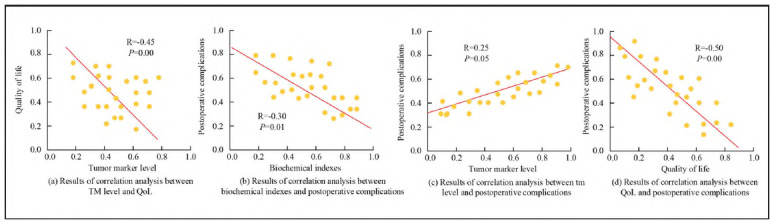
Correlation analysis results.

This study conducted a correlation analysis to explore the potential relationship between TML, biochemical indicators, postoperative QoL, and patient complications. The correlation between multiple variables was evaluated using Spearman's method, as shown in Figure 5. In Figure 5 (a), there was a significant negative correlation between the decrease of TMs and the improvement of QoL (R=-0.45, *P*=0.00). In Figure 5 (b), the improvement of biochemical indicators such as ALT, AST, and ALP in patients after treatment was inactively related to the incidence of POC (R=-0.30, *P*=0.01). In Figure 5 (c), the direct correlation between TML and POC was weak (*P*=0.05). In Figure 5 (d), multiple dimensions of QoL were inactively connected to the incidence of POC (R=-0.50, *P*=0.00).

### PPS

To evaluate in detail the pain relief effect of IORT-DT on patients with LPT, this study used NRS to record the pain levels of both groups. NRS divided pain levels into 0-10 points, with 0 denoting no pain and 10 denoting unbearable severe pain. Table 6 shows the specific data. In pre-treatment, there was *P*=0.76 in pain scores between EG and CG patients. At different time points after treatment, the pain score of EG was lower (*P*<0.05), and the pain score showed a decreasing trend overtime. In addition, the pain relief rate of EG was relatively fast, with significant pain relief observed within one week after treatment, while the pain relief of CG was relatively slow.

**Table 6:** Comparison of pain scores between EG and CG.

Time Point	EG (n = 95)	CG (n=95)	t	*P*
Pre-treatment	6.8±2.1	6.9±2.0	0.31	0.76
1 -week post-treatment	4.2±1.8	5.6±2.2	4.52	0.00
1-month post-treatment	3.1 ±1.5	4.3±1.9	4.06	0.00
3 months post-treatment	2.4±1.2	3.5±1.7	4.87	0.00
6 months post-treatment	2.0±1.0	2.9±1.4	4.21	0.00

### Psychological state assessment

To evaluate the impact of IORT-DT on the psychological status of patients with LPT, HADS was taken to assess the anxiety and depression status of both groups. HADS includes the anxiety subscale (HADS-A) and depression subscale (HADS-D). Each sub-scale contains 7 items and uses a four-point rating system ranging from 0 to 3. The higher the score, the more severe the symptoms (Table 7). In pretreatment, there was *P*>0.05 in scores between EG and CG patients on the HADS-A and HADS-D sub-scales. After treatment, the HADS-A and HADS-D scores of EG were lower (*P*<0.05), and the scores showed a decreasing trend overtime. In addition, the psychological state of EG improved rapidly, with significant reductions in anxiety and depression symptoms observed within one week after treatment, while CG improved more slowly.

**Table 7:** Comparison of psychological status between the two groups.

Time Point	Sub-scale	EG (n=95)	CG (n=95)	t	P
Pre-treatment	HADS-A	11.2±4.3	11.5 ±4.1	0.42	0.67
	HADS-D	9.8±3.9	10.1 ±3.7	0.48	0.63
1 -week post-treatment	HADS-A	8.4±3.5	10.3±3.8	3.31	0.01
	HADS-D	7.2±3.2	8.9±3.5	3.02	0.01
1-month post-treatment	HADS-A	6.8±2.9	8.6±3.2	3.67	0.00
	HADS-D	5.9±2.7	7.5±3.0	3.45	0.00
3 months post-treatment	HADS-A	5.6±2.4	7.2±2.8	3.91	0.00
	HADS-D	5.1 ±2.3	6.7±2.6	3.72	0.00
6 months post-treatment	HADS-A	4.8±2.1	6.3±2.4	3.84	0.00
	HADS-D	4.5±2.0	6.0±2.3	3.68	0.00

## Discussion

Hepatopancreatic tumours are malignant tumours with extremely high mortality rates world-wide. Due to their insidious early symptoms and high mortality rates, they have long been a difficult problem that the global medical community urgently needs to overcome [20] [21]. Patients often miss the best treatment opportunity when their condition has advanced to the late stage by the time of diagnosis. With the advancement of medical technology, especially the breakthrough progress of IORT technology, new methods have been provided for treating LPT [22] [23]. IORT, with its precise targeting of tumour tissue and minimising damage to healthy tissue, significantly improves the safety margin and efficacy expectations of treatment [24]. On this basis, the integration of cutting-edge technologies such as deep learning and image guidance has made its localisation and treatment process more refined and personalised, further consolidating its position in the comprehensive treatment of LPT [25] [26]. Based on this background, this study innovatively focuses on the IORT-DT. Through systematic analysis of its therapeutic effect in patients with LPT, the aim is to reveal new mechanisms under the synergistic effect of the two and open up more efficient and safe treatment pathways for clinical practice.

Research has shown that after receiving IORT-DT, the average level of tumour marker AFP in EG patients decreased by 16.2 ng/mL, nearly twice as high as the 8.6 ng/mL in CG (*P*<0.05). The significant difference was validated by the t-test, indicating that IORT-DT has a substantial advantage in reducing AFP levels. Similarly, the decrease in CA19-9 levels showed a similar trend, with a mean reduction of 74.7 U/mL in EG and only 39.9 U/mL in CG. This trend (*P*<0.05) strongly suggested the effectiveness of combination therapy in inhibiting the growth of TMs. Compared with existing literature, this result was consistent with Midya Jayarsee Chakraborty et al.'s [27] research. They utilised deep neural networks for computer-aided diagnosis of liver tumours, emphasising the importance of precise diagnosis in improving treatment outcomes. This study further improved the accuracy and effectiveness of treatment by combining IORT with drug therapy. Meanwhile, after treatment, the average levels of ALT, AST, and ALP in EG patients decreased by 9.7 U/L, 11.8 U/L, and 21.8 U/L, respectively, while the corresponding indicators in CG showed a smaller decrease. The results were statistically supported by t-tests (*P*<0.05), indicating the superiority of the combination therapy in reducing liver injury. Renal function indicators such as serum creatinine and urea nitrogen also showed a similar improvement trend, further verifying the safety of this treatment regimen. Additionally, the recurrence and metastasis rates of EG were lower than those of CG, indicating that combination therapy also has a noticeable effect in reducing the risk of tumour recurrence and metastasis. It was also found that although there was no significant difference in common AR in both groups, EG had a lower incidence of ARs in radiation dermatitis and infection. This may be related to the precision of IORT and the specificity of drug treatment. This finding was consistent with Zhang et al.'s [28] research on absorbed dose estimation in molecular radiotherapy, which pointed out that accurate dose distribution can help improve treatment safety [29] [30].

Zhang and colleagues [31] investigated the efficacy of IORT combined with adjuvant chemotherapy in treating pTSNOMO rectal adenocarcinoma. They found that it provided comparable local control, overall survival, and disease-free survival to external beam radiotherapy (EBRT) while reducing acute toxicities. In contrast, our study focused on hepatic and pancreatic tumours, integrating IORT with targeted therapy and immunomodulators instead of chemotherapy. While both studies highlight the benefits of IORT, our approach addresses the higher metastatic potential of LPTs by incorporating systemic drug therapy to enhance tumour suppression, reduce recurrence, and improve patient outcomes. This comprehensive treatment strategy offers a novel alternative to traditional lORT-based regimens, potentially expanding its clinical applicability beyond rectal cancer.

Edoo and colleagues [32] evaluated the diagnostic utility of AFF) CA19-9, and CEA in detecting primary hepatocellular carcinoma (PHC). They found that while AFP alone had a sensitivity of 63.3% and specificity of 80.8%, combining it with CA19-9 and CEA increased specificity to 100% but significantly reduced sensitivity. Their study concluded that AFP remains the most reliable biomarker for PHC screening, as the combined markers did not offer a diagnostic advantage. In contrast, our study focused on the therapeutic implications of AFP and CA19-9 levels in liver and pancreatic tumours following intraoperative radiotherapy (IORT) combined with drug therapy, demonstrating that a more significant reduction in these markers correlated with improved treatment outcomes, lower recurrence rates, and enhanced quality of life. While both studies highlight the clinical significance of AFP and CA19-9, our research uses these biomarkers as indicators of therapeutic response rather than primary diagnostic tools.

He and colleagues [33] conducted a metaanalysis comparing intraoperative radiotherapy (IORT) as a tumour-bed boost combined with whole breast irradiation (WBI) versus conventional radiotherapy in early-stage breast cancer. Their findings showed that while IORT boost+WBI did not significantly reduce local recurrence rates, it significantly improved disease-free survival (DFS) and reduced distant metastasis rates (DMR), particularly with electron boost. Additionally, IORT demonstrated similar cosmetic and safety profiles compared to conventional radiotherapy. In contrast, our study investigated IORT combined with drug therapy (IORT-DT) in liver and pancreatic tumours, focusing on its effect on tumour marker reduction (AFF) CA19-9), recurrence rates, survival outcomes, and quality of life. While both studies support the efficacy and safety of IORT, our research expands its application by integrating systemic drug therapy to enhance tumour control and reduce metastasis in aggressive malignancies, demonstrating a novel multi-modal approach beyond local radiotherapy strategies.

Regarding exploring QoL and psychological status, the study found that EG patients' average physical function score increased by 7.2 points (*P*<0.05). The role function score rose 9.6 points (*P*<0.05), exceeding CG. This result indicated that combination therapy significantly improved patients' daily living abilities and social participation. In addition, the emotional function score of EG patients also showed significant improvement (*P*<0.05), which may be related to their positive expectations of treatment outcomes and psychological support during treatment. This result confirmed the combination therapy's effectiveness in improving patients' QoL and emphasised the importance of psychological intervention in tumour treatment. Compared with the study by Wang et al. [29], they used a multi-modal imaging system to image the pancreatic biliary duct, aiming to improve the accuracy of diagnosis. This study enhanced treatment efficacy through a combination therapy approach and improved patients' QoL and psychological state, providing a new perspective for the comprehensive treatment of LPT. In addition, the correlation analysis results showed that the decrease in AFP and CA19-9 levels was significantly negatively correlated with improved scores in dimensions such as physical function and role function (R=- 0.45, *P*=0.00). This discovery emphasised the importance of TMs as indicators for evaluating treatment efficacy. Meanwhile, the improvement of biochemical indicators was significantly negatively correlated with the incidence of POC (R=-0.30, *P*=0.01), further confirming the effectiveness of the combination therapy in reducing treatment-related complications. This finding was coherent with the research of Li et al. [30], who used a dual self-supervised learning framework to model pancreatic segmentation and also emphasised the key role of precise segmentation in evaluating treatment efficacy. This study achieved a reduction in TMs and a significant improvement in QoL through combination therapy, providing new evidence for assessing the treatment efficacy of LPT. Finally, the psychological state assessment data showed that the scores of EG patients on the HADS-A and HADS-D sub-scales were significantly lower than those of CG, and this difference remained consistent at all time points after treatment (*P*<0.05). The above data not only demonstrated the effectiveness of combination therapy in alleviating patients' anxiety and depression symptoms but also provided strong support for the importance of psychological intervention in tumour treatment [31] [32] [33].

Overall, this paper systematically explored the IORT-DT efficacy in treating LPT firstly. Research data showed that this combination therapy had significant advantages in reducing TML, improving LKF, reducing recurrence and metastasis rates, improving survival and QoL, as well as reducing POC and ARs. This discovery not only provided new effective strategies for the treatment of LPT but also provided important references for future related research. Meanwhile, this study emphasised the importance of precise diagnosis and personalised treatment in tumour treatment, providing a new perspective for the comprehensive treatment of LPT. Future research can further explore the synergistic mechanism between IORT and different drug treatment regimens and the application effect of this combination therapy in patients with varying types of LPT to provide more precise and effective treatment strategies for clinical practice.

## Conclusion

The study demonstrates that IORT-DT is an effective treatment for liver and pancreatic tumours LPT, significantly reducing AFP and CA19-9 levels, improving liver and kidney function, lowering recurrence rates, and enhancing survival outcomes compared to conventional treatments. IORT-DT patients experienced better quality of life, reduced pain, and fewer postoperative complications. These findings suggest that IORT-DT offers a promising, comprehensive approach for LPT treatment. Further research is needed to optimise treatment protocols and confirm long-term benefits.

## Dodatak

### Conflict of interest statement

All the authors declare that they have no conflict of interest in this work.
